# Supplementary material for: Unexpected functional implication of a stable succinimide in the structural stability of Methanocaldococcus jannaschii glutaminase
Source: Nat Commun. 2016 Sep 28;7:12798. doi: 10.1038/ncomms12798 (PMC5052720; doi:10.1038/ncomms12798)
Supplement: Supplementary Information — Supplementary Figures 1-12 and Supplementary Table 1 [file ncomms12798-s1.pdf]

Supplementary information

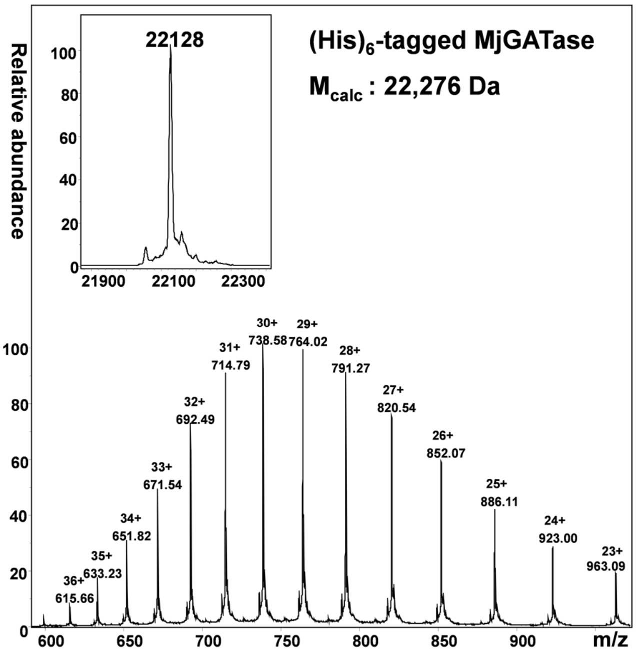

Supplementary figure 1. Mass of $(His)_6$-tagged MjGATase shows loss of 17 Da over the expected mass. This indicates that the loss of an NH_3 molecule is not an artifact arising from the purification procedure. ESI-MS of $(His)_6$-tagged MjGATase (M_{calc} 22,276 Da). Inset: deconvoluted spectrum that shows a M_{obs} of 22,128 Da. This mass indicates loss of an NH_3 molecule and the N-terminal Met residue from the protein sequence. The M_{calc} of 22,276 Da of $(His)_6$-tagged MjGATase is due to presence of extra residues (Met, Ala, Ser, His_6 , Gly and Ser) derived from the vector sequence and the BamHI restriction site

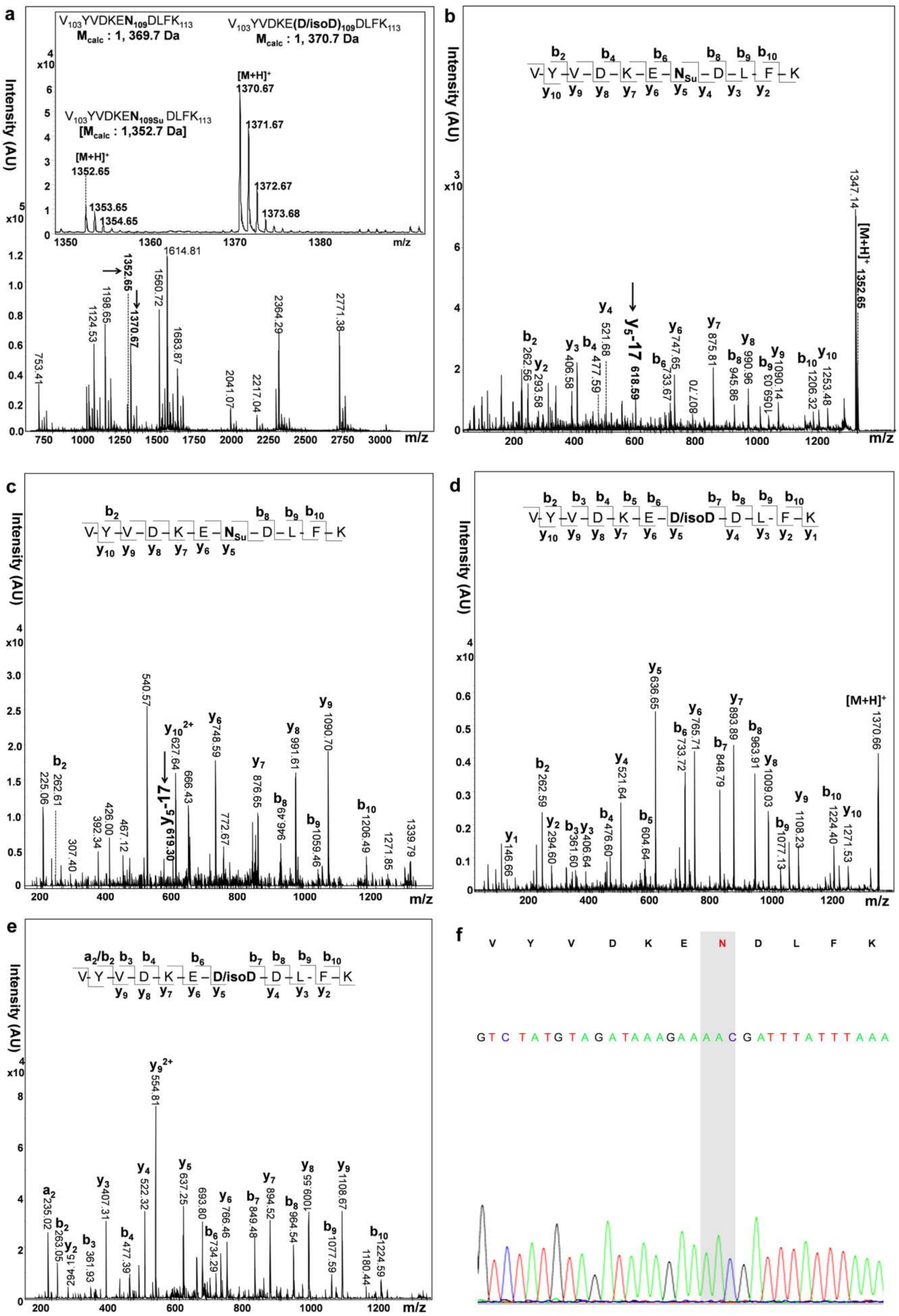
a
V103YVDKEN109DLFK113
Mcalc : 1, 369.7 Da
V103YVDKE(D/isoD)109DLFK113
Mcalc : 1, 370.7 Da
V103YVDKEN109Su DLFK113
[Mcalc : 1,352.7 Da]
[M+H]+
1352.65
1353.65
1354.65
1370.67
1371.67
1372.67
1373.68
Intensity (AU)
m/z
753.41
1124.53
1198.65
1352.65
1370.67
1560.72
1614.81
1683.87
2041.07
2217.04
2364.29
2771.38
b
V-Y-V-D-K-E-NSu-D-L-F-K
y5-17
618.59
[M+H]+
1347.14
1352.65
c
V-Y-V-D-K-E-NSu-D-L-F-K
540.57
y5-17
619.30
y10 2+
627.64
d
V-Y-V-D-K-E-D/isoD-D-L-F-K
[M+H]+
1370.66
e
V-Y-V-D-K-E-D/isoD-D-L-F-K
y9 2+
554.81
f
V Y V D K E N D L F K
GTCTATGTAGATAAAGAAAACGATTTATTTAAA

Supplementary figure 2. N109 in MjGATase is modified to succinimide.
(a) MALDI-MS of in-gel trypsin-digested MjGATase where peaks corresponding to peptide fragment with succinimide and the hydrolyzed product are highlighted by the arrows. Inset: zoom-in of the peaks corresponding to 1,352.7 Da and 1,370.7 Da. These masses are 17 Da lower and 1 Da more than the expected mass of $V_{103}YVDKE\mathbf{N_{109}}DLFK_{113}$. Fragmentation of 1,352.7 Da peptide by **(b)** MALDI-MS/MS and **(c)** CID-MS/MS. Highlighted by an arrow is the first y ion that is 17 Da lower than the expected mass. Fragmentation of 1,370.7 Da peptide by **(d)** MALDI-MS/MS and **(e)** CID-MS/MS. These peptides were obtained by in-gel trypsin digestion of MjGATase. Mass of 1,352.7 Da corresponds to the peptide, $V_{103}YVDKE\mathbf{N_{Su}}DLFK_{113}$ with succinimide at position 109 while 1,370.7 Da arises from hydrolysis of succinimide. **(f)** A portion of DNA sequence electropherogram of MjGATase showing the gene sequence that yields a peptide with the expected sequence of $V_{103}YVDKE\mathbf{N_{109}}DLFK_{113}$ (M_{calc} 1,369.7 Da). M_{obs} of 1,352.7 Da is due to a post-translational modification of N109 to succinimide.

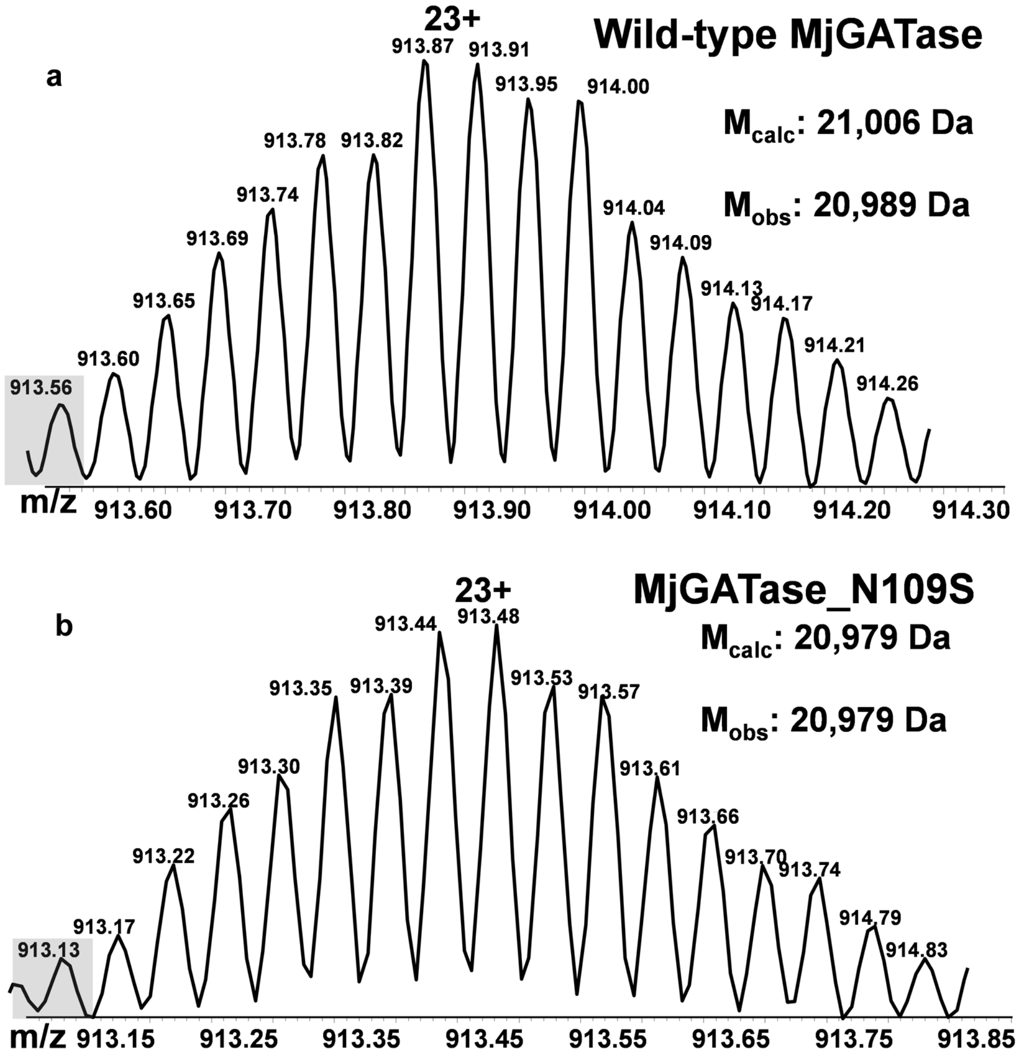

Supplementary figure 3. ESI-MS recorded on an Orbitrap mass spectrometer in monoisotpic mode corroborates the presence of succinimide in MjGATase. ESI-MS of **(a)** Wild-type MjGATase and **(b)** N109S_MjGATase. Figure shows one of the most abundant charge states (+23) that indicate the isotopic resolution of the mass spectrum. First isotope is highlighted in grey. Wild-type MjGATase shows a mass of 20,989 Da, which is 17 Da lower than the monoisotpic M_{calc} of 21,006 Da while N109S_ MjGATase shows M_{obs} of 20,979 Da in agreement with the monoistopic M_{calc} of 20,979 Da.

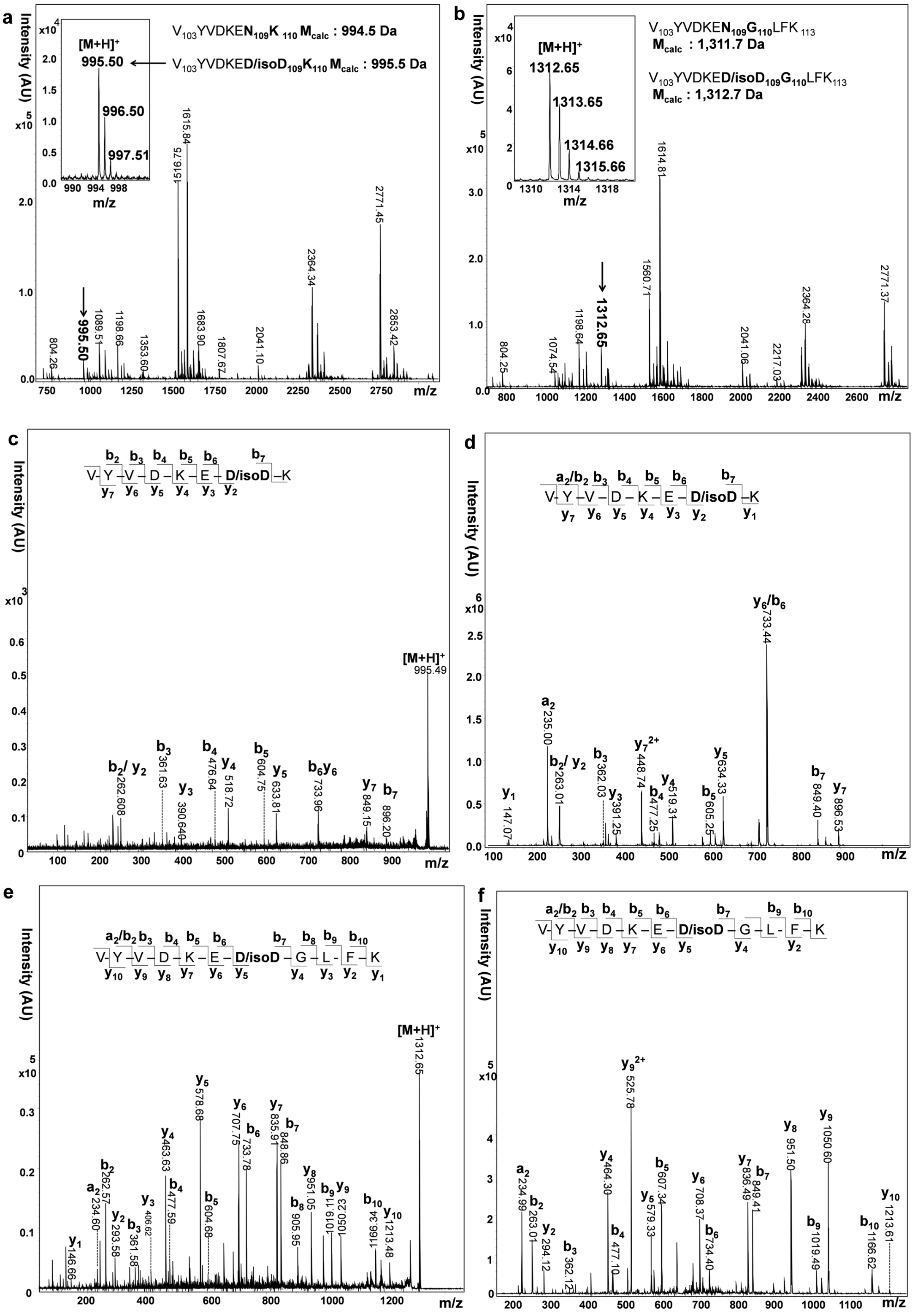
a
Intensity (AU)
[M+H]+
995.50
996.50
997.51
m/z
V103YVDKEN109K110 Mcalc : 994.5 Da
V103YVDKED/isoD109K110 Mcalc : 995.5 Da
804.26
995.50
1089.51
1198.66
1353.60
1519.75
1615.84
1683.90
1807.67
2041.10
2364.34
2771.45
2853.42
b
Intensity (AU)
[M+H]+
1312.65
1313.65
1314.66
1315.66
m/z
V103YVDKEN109G110LFK113
Mcalc : 1,311.7 Da
V103YVDKED/isoD109G110LFK113
Mcalc : 1,312.7 Da
804.25
1074.54
1198.64
1312.65
1560.71
1614.81
2041.06
2217.03
2364.28
2771.37
c
Intensity (AU)
V-Y-V-D-K-E-D/isoD-K
[M+H]+
995.49
262.608
361.63
390.640
476.64
518.72
604.75
633.81
733.96
849.15
896.20
m/z
d
Intensity (AU)
V-Y-V-D-K-E-D/isoD-K
y6/b6
733.44
a2
235.00
263.01
362.03
391.25
448.74
477.25
519.31
605.25
634.33
849.40
896.53
147.07
m/z
e
Intensity (AU)
V-Y-V-D-K-E-D/isoD-G-L-F-K
[M+H]+
1312.65
146.66
234.60
262.57
293.58
361.58
406.62
463.63
477.59
578.68
604.68
707.75
733.78
835.91
848.86
905.95
951.05
1019.11
1050.23
1166.34
1213.48
m/z
f
Intensity (AU)
V-Y-V-D-K-E-D/isoD-G-L-F-K
y9 2+
525.78
234.99
263.01
294.12
362.12
464.30
477.10
579.33
607.34
708.37
734.40
836.49
849.41
951.50
1019.49
1050.60
1166.62
1213.61
m/z

Supplementary figure 4. D110 is essential for stabilizing succinimide in MjGATase. MALDI-MS of trypsin-digested samples of **(a)** MjGATase_D110K and **(b)** MjGATase_D110G where peaks corresponding to peptide fragments (995.5 Da and 1,312.7 Da) with hydrolyzed succinimide are highlighted by arrows. Insets: zoom-in of peaks corresponding to 995.5 Da in MjGATase_D110K and 1,312.7 Da in MjGATase_D110G. The observed masses are 1 Da more than the expected masses of peptides V_{103}YVDKE$\mathbf{N_{109}K_{110}}$ and V_{103}YVDKE$\mathbf{N_{109}G_{110}}$LFK_{113}. Fragmentation of 995.5 Da peptide by **(c)** MALDI-MS/MS and **(d)** CID-MS/MS. Fragmentation of 1,312.7 Da peptide by **(e)** MALDI-MS/MS and **(f)** CID-MS/MS. Panels c, d, e and f show fragment ion masses that indicate the presence of Asp/isoAsp at position 109 arising from hydrolysis of the succinimidyl moiety in the two mutants.

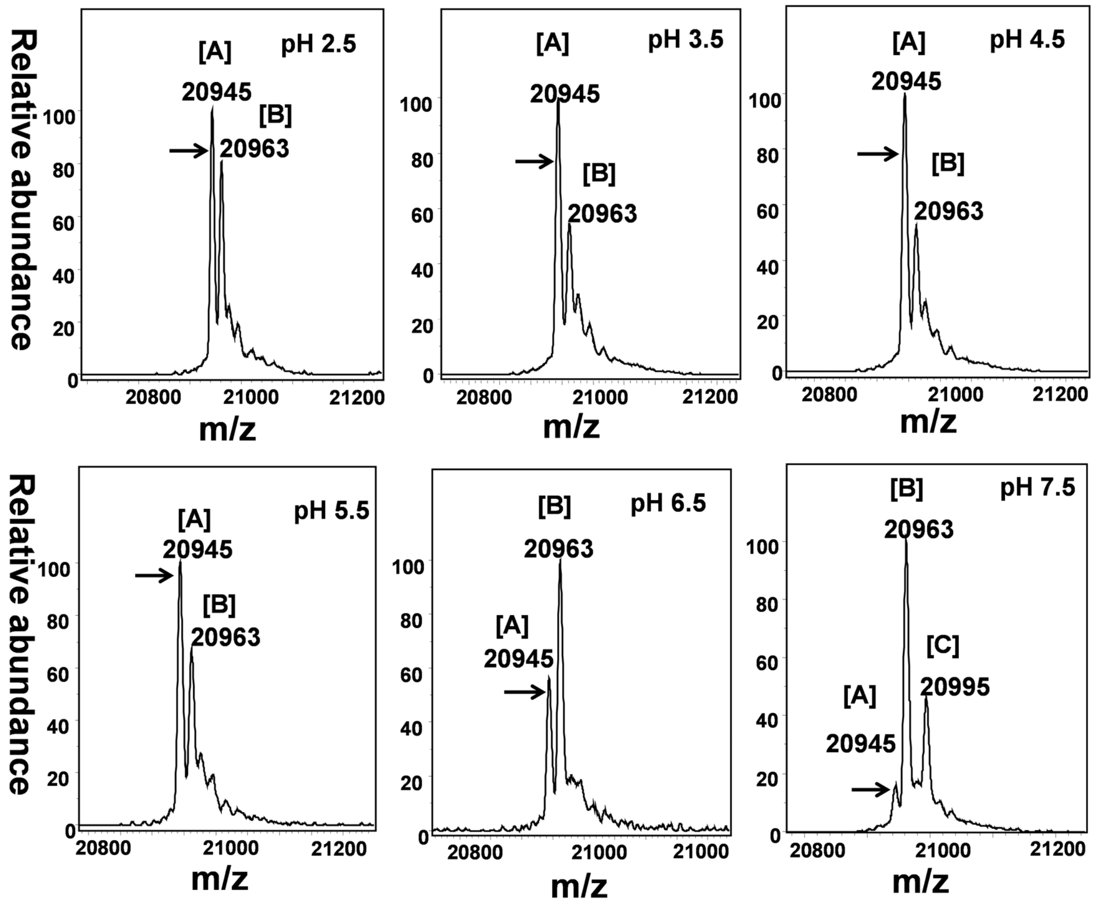

Supplementary figure 5. MjGATase_D110G forms and retains succinimide at low pH that rapidly hydrolyzes at high pH. Deconvoluted ESI-MS of MjGATase_D110G recorded after 6 hr of incubation in solutions of indicated pH are shown in the individual panels. The peak in the mass spectra corresponding to protein species with succinimide is indicated by an arrow.

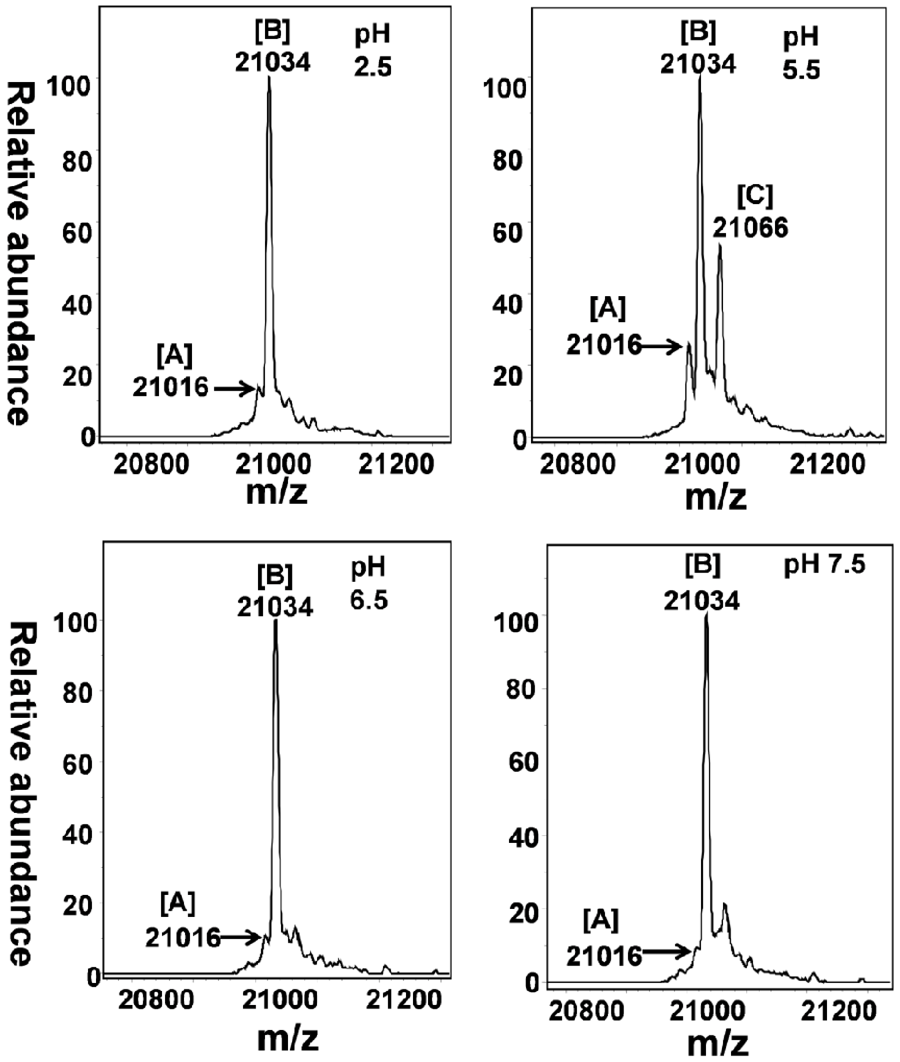

Supplementary figure 6. Similar to MjGATase_D110G, MjGATase_D110K also forms and retains succinimide at low pH albeit with lower efficiency. Deconvoluted ESI-MS of MjGATase_D110K recorded after 6 hr of incubation in solutions of indicated pH are shown in the individual panels. The peak in the mass spectra corresponding to species with succinimide is indicated by an arrow.

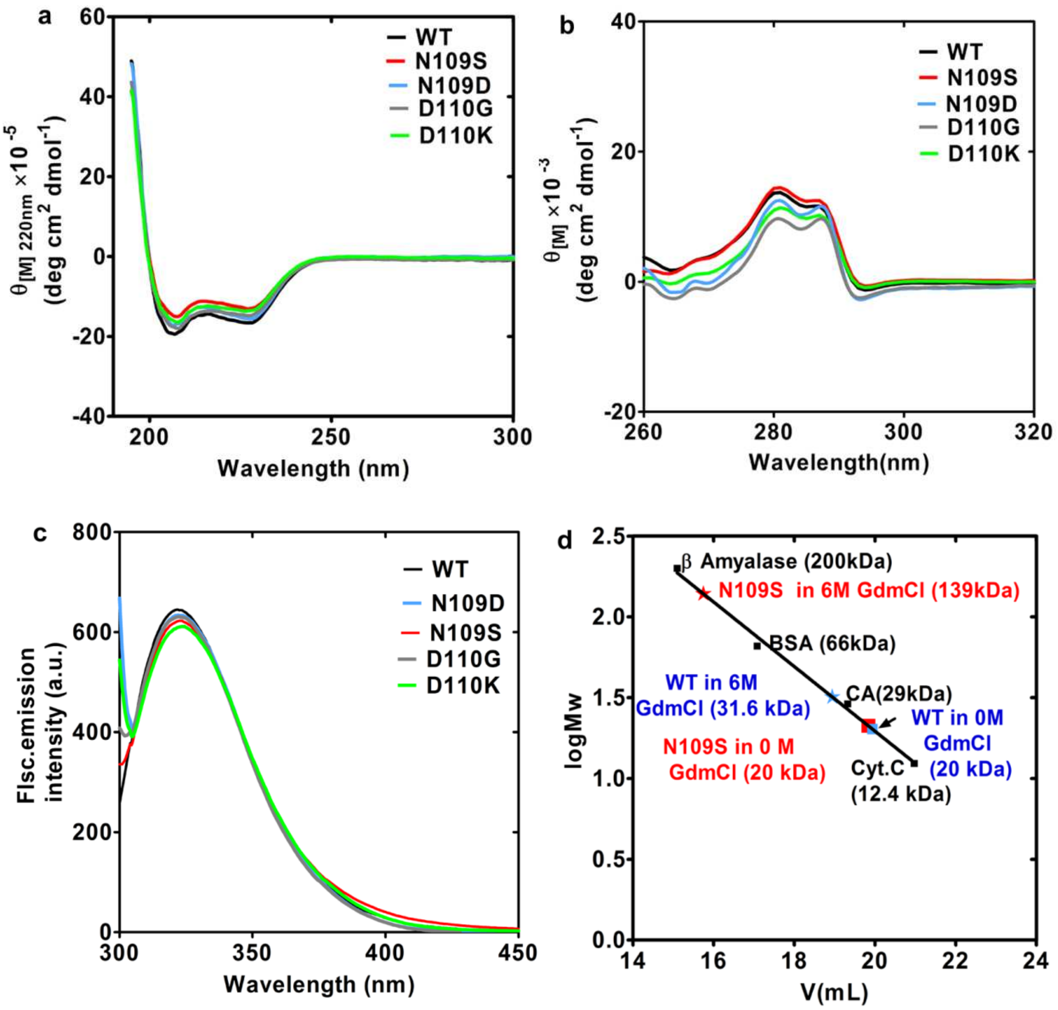

Supplementary figure 7. Presence or absence of succinimide does not affect the overall structure of MjGATase at 25 ℃| (a) Far-UV CD, **(b)** Near-UV CD and **(c)** tryptophan fluorescence spectra of WT and mutants of MjGATase recorded at 25 ℃. Near identical spectra of enzymes with succinimide (WT_{Su} and MjGATase_N109D_{Su}) and mutants (MjGATase_N109S, D110G and D110K) having little or no succinimide indicate that absence/presence of succinimide does not perturb the overall structure of MjGATase at 25 ℃. **(d)** Calibration curve of log molecular weight (M_w) versus elution volume. Highlighted in red and cyan squares, respectively are the elution volumes of WT MjGATase and MjGATase_ N109S in the absence of chaotrope while red star and cyan star, respectively indicate elution volumes of the two proteins in the presence of 6 M GdmCl.

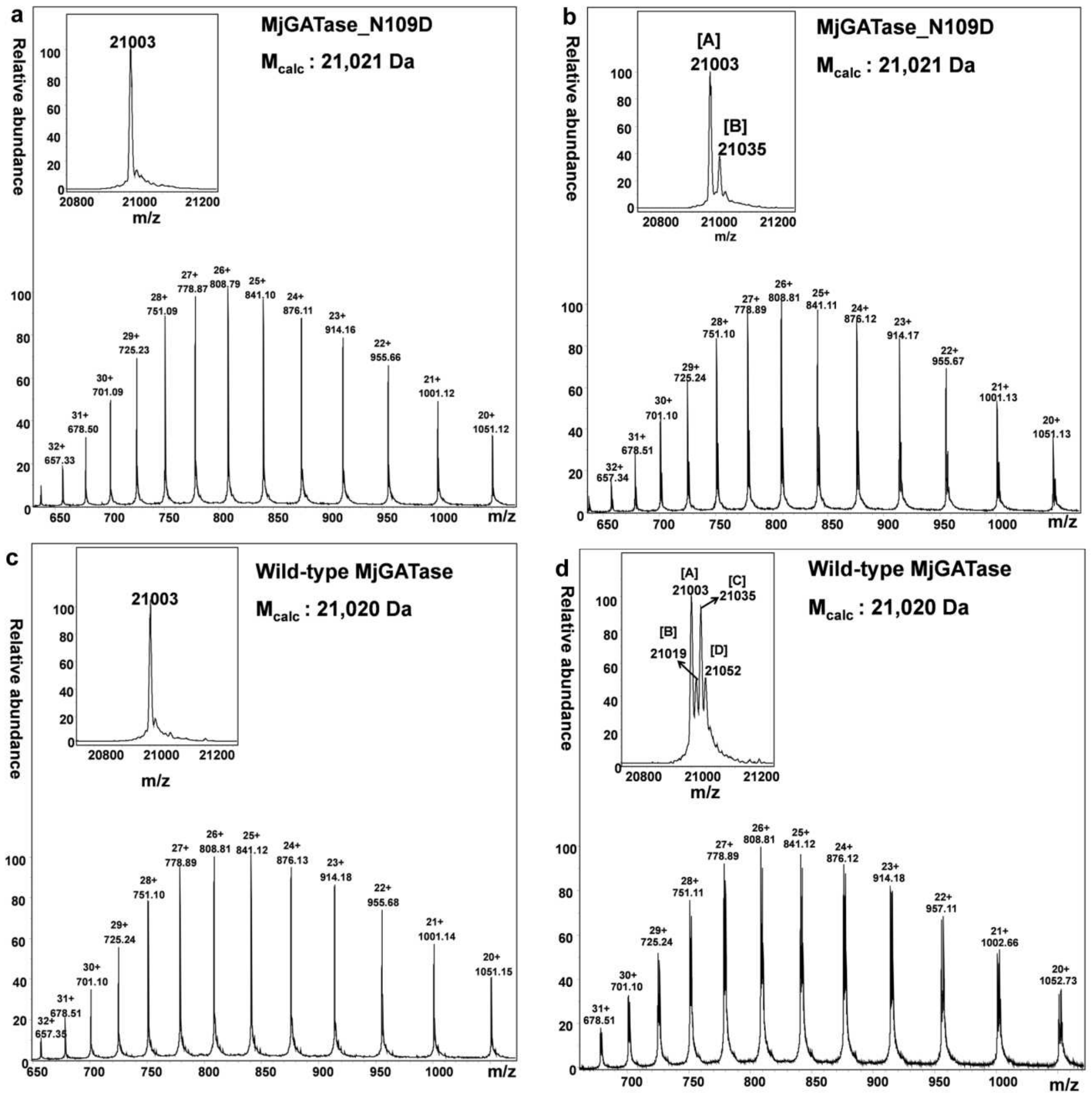

Supplementary figure 8. Stability of succinimide in MjGATase_N109D$_{Su}$ and WT$_{Su}$. LC-ESI-MS of MjGATase_ N109D (M_{calc} 21, 021 Da) recorded after incubation **(a)** at 100 ℃ for 15 min and **(b)** in 8 M GdmCl for 12 hr at 37 ℃. Inset to panel a shows M_{obs} of 21,003 Da. This mass corresponds to MjGATase _N109D$_{Su}$. Inset to panel b shows [A] of M_{obs} 21,003 Da and [B] of M_{obs} 21,035 Da. The mass of 21,003 corresponds to MjGATase_N109D$_{Su}$ while 21,035 Da indicates N109D$_{Su}$ along with the addition of two oxygen atoms arising from methionine oxidation. **(c)** LC-ESI-MS of WT$_{Su}$ recorded after incubation in 0.1 N HCl at 37 ℃ for 12 hr. Inset: deconvoluted ESI-MS showing presence of succinimide, M_{obs} 21,003 Da. **(d)** LC-ESI-MS of WT$_{Su}$ recorded after incubation in 2 M NH_2OH at 37 ℃ for 12 hr. Inset: deconvoluted ESI-MS s howing [A] of M_{obs} 21,003 Da, [B] of M_{obs} 21,019 Da, [C] of M_{obs} 21,035 Da and [D] of 21,052 Da. The mass of 21,003 Da corresponds to WT$_{Su}$ while M_{obs} 21,019 Da and 21,035 Da correspond to WT$_{Su}$ with addition of one and two oxygen atoms, respectively. The mass of 21,052 Da could be assigned to either oxidized WT$_{Su}$ or a hydroximic acid derivate.

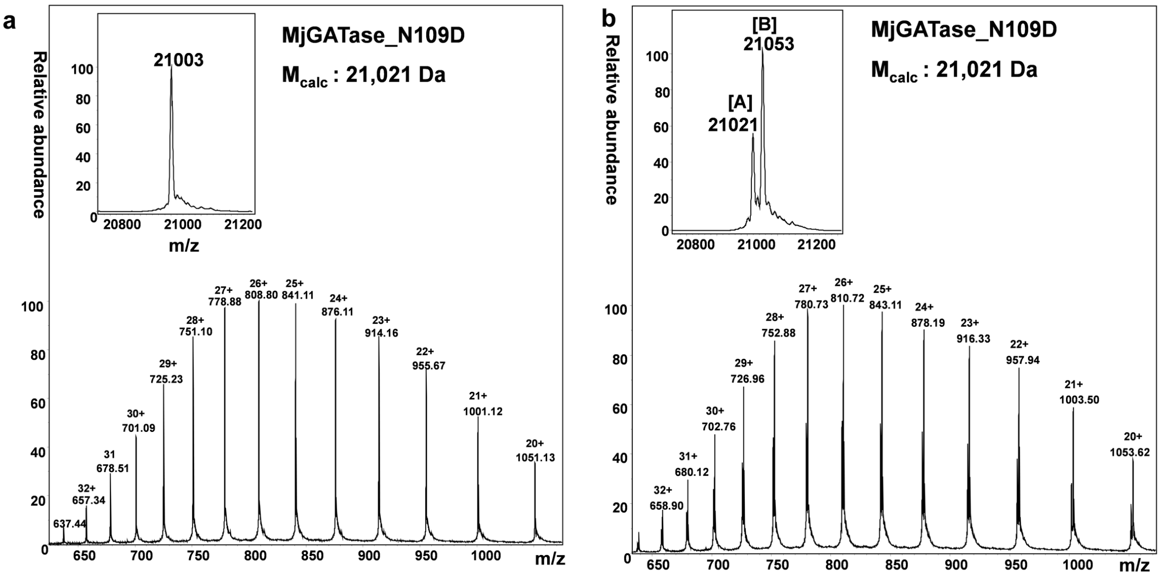

Supplementary figure 9. As in WT MjGATase, succinimide in MjGATase_N109D is also hydrolyzed at pH 10.5. LC-ESI-MS of MjGATase_N109D (M_{calc} 21,021 Da). **(a)** Spectrum recorded after incubation in a solution of pH 2.5. Inset: deconvoluted spectrum M_{obs} 21,003 Da. This mass corresponds to the mass of MjGATase_N109D$_{su}$. **(b)** Spectrum recoded after incubation in a solution of pH 10.5. Inset: deconvoluted spectrum that shows [A] of M_{obs} 21,021 Da which is indicative of the absence of succinimide and [B] of M_{obs} 21,053 Da indicates hydrolysis of succinimide along with the addition of two oxygen atoms. At both pH values, the incubation time was for 12 hr at 37 ℃.

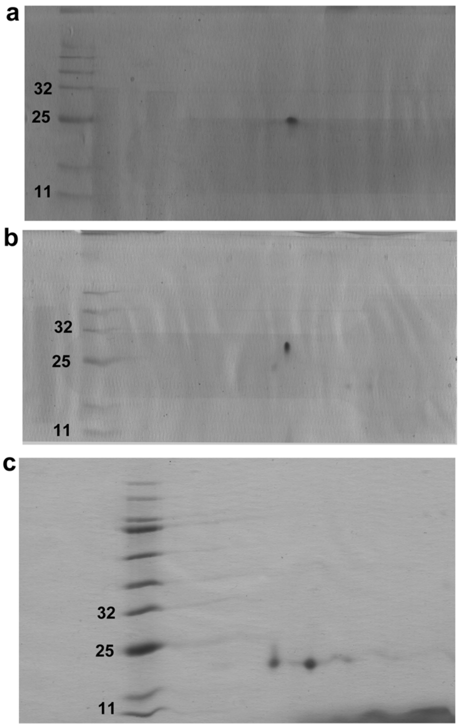

Supplementary figure 10. Two-dimensional gel electrophoresis of MjGATase corroborates hydrolysis of succinimide at high pH. MjGATase preincubated in a solution of pH 10.5 has mobility on two-dimensional gel electrophoresis that is distinct from that of the protein incubated at pH 7.4. SDS-PAGE after IEF of MjGATase **(a)** Preincubated at pH 7.4 **(b)** pH 10.5 and **(c)** mixture of a and b. All samples were buffer exchanged to pH 7.4 prior to IEF. Panels a and b with one spot indicate the presence of a single species with one pI while the mixture in panel c showing two spots indicates the presence of two species. The spot with higher pI value corresponds to a protein with succinimide while the one of lower pI is MjGATase with hydrolyzed succinimide. The left most lane is molecular weight marker proteins with relevant masses indicated in kDa.

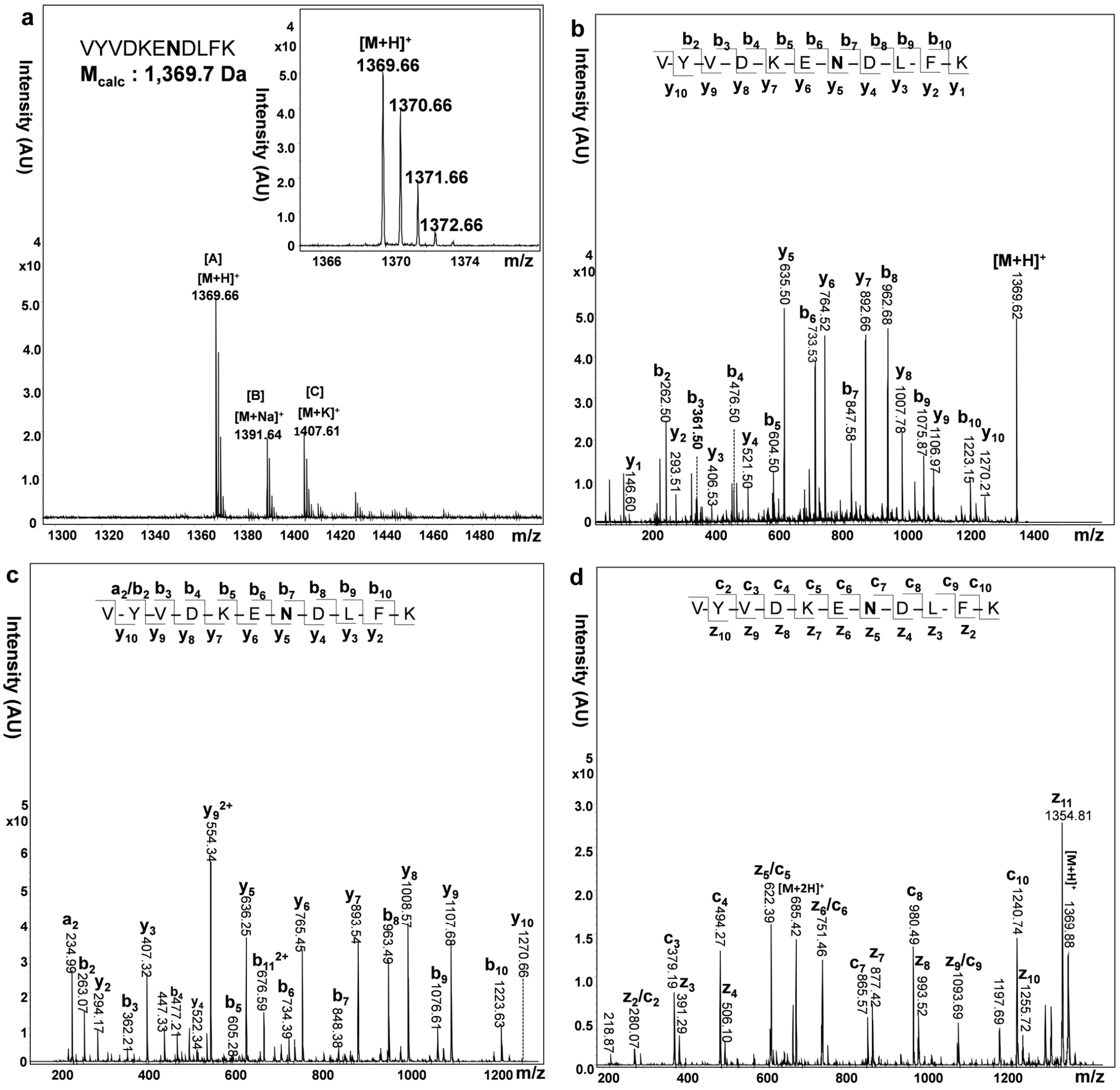

Supplementary figure 11. Synthetic peptide, VYVDKENDLFK does not form succinimide or undergo deamidation. (a) MALDI-MS of VYVDKENDLFK (M_{calc} 1,369.7 Da). Spectrum shows M_{obs} of [A] 1,369.7 Da, [B] 1,391.6 Da and [C] 1,407.6 Da. Mass of 1,369.7 Da corresponds to the mass of the native peptide while 1,391.6 Da and 1,407.61Da correspond to the mass of VYVDKENDLFK as mono sodium and mono potassium adducts, respectively. Fragmentation by **(b)** MALDI-MS/MS, **(c)** CID-MS/MS and **(d)** ETD-MS/MS. Fragmentation by MALDI and CID-MS/MS of synthetic peptide yields the sequence VYVDKENDLFK suggesting the absence of succinimide in the peptide fragment. ETD-MS/MS shows the absence of diagnostic c+57 and z-57 ions. This confirms that the synthetic peptide does not form succinimide.

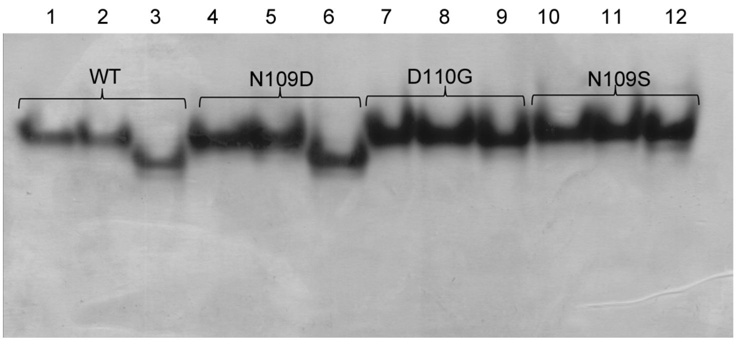

Supplementary Figure 12. Native PAGE supports hydrolysis of succinimide at high pH. Native-PAGE of WT (lane 1-3), MjGATase_N109D (lane 4-6), MjGATase_D110G (lane 7-9) and MjGATase_N109S (lane 10-12). The protein samples were incubated in a solution of pH 2.5 (lanes 1, 4, 7 and 10), 7.4 (lanes 2, 5, 8 and 11), and 10.5. (lanes 3, 6, 9 and 12) for 12 hr at 37 ℃ . The pH of all the samples was adjusted to 7.4 before electrophoresis.

Supplementary Table 1. List of primers used for cloning and site-directed mutagenesis. Bases which were changed to incorporate restriction site and the desired mutation are highlighted in bold.

MjGATase_N109D	Fw-5′-GGTCTATGTAGATAAAGAA**G**A**T**GATTTATTTAAAAACGTTCCAAG-3'<br>Rev-5′-CTTGGAACGTTTTTAAATAA**A**T**C**ATCTTCTTTATCTACATAGACC-3'
MjGATase_N109S	Fw-5′-GGTCTATGTAGATAAAGAAA**G**CGATTTATTTAAAAACGTTCC-3'<br>Rev-5′-GGAACGTTTTTAAATAAATCG**C**TTTCTTTATCTACATAGACC-3'
MjGATase_D110G	Fw-5′-GGTCTATGTAGATAAAGAAAACG**GC**TTATTTAAAAACGTTCCAAGAGAG-3'<br>Rev-5'-CTCTCTTGGAACGTTTTTAAATAA**GC**CGTTTTCTTTATCTACATAGACC3'
MjGATase_D110K	Fw-5'-GGTCTATGTAGATAAAGAAAAC**A**A**A**TTATTTAAAAACGTTCCAAGAG 3'<br>Rev-5'-CTCTTGGAACGTTTTTAAATAA**T**T**T**GTTTTCTTTATCTACATAGACC-3'
$(His)_6$-tagged MjGATase	Fw-5'GC**GGATCC**ATGATTGTTATCTTAGACAACGGAGGG3'<br>Rev-5'GCA**GAGCTC**TTATTCAAATTTGTAACCACAAACTTTACAAAAG3'
